# Comparative efficacy of Chinese herbal injections for treating chronic heart failure: a network meta-analysis

**DOI:** 10.1186/s12906-018-2090-3

**Published:** 2018-01-31

**Authors:** Kai-Huan Wang, Jia-Rui Wu, Dan Zhang, Xiao-Jiao Duan, Meng-Wei Ni

**Affiliations:** Department of Clinical Pharmacology of Traditional Chinese Medicine, School of Chinese Materia Medica, Beijing University of Chinese Medicine, Beijing, 100102 China

**Keywords:** Network meta-analysis (NMA), Chronic heart failure (CHF), Chinese herbal injection (CHI)

## Abstract

**Background:**

On account of deterioration of chronic heart failure (CHF) and extensive exploration of Chinese herbal injections (CHIs), we performed a network meta-analysis to investigate the efficacy of CHIs (Huangqi injection, Shenfu injection, Shengmai injection, Shenmai injection, Shenqi Fuzheng injection, Yiqifumai injection) on the basis of western medicine (WM) treatment in CHF.

**Methods:**

Literature search was conducted in Embase, the Cochrane Library, Pubmed, Chinese Biological Medicine Database, China National Knowledge Infrastructure, Wanfang Database, Chinese Scientific Journal Database from inception to June 12nd 2017, and study selection was abided by a prior eligible criteria.

**Results:**

Ultimately, a total of 113 randomized controlled trials (RCTs) were enrolled. The clinical data of the effective clinical rate, left ventricular ejection fraction, cardiac output and others outcomes was estimated by Stata software and Winbugs software. Risk of bias was assessed by Cochrane Collaboration’s tools. Integrating the each outcome’s results, a combination of Shengmai injection/Shenmai injection and WM obtain a first rank in most outcomes, particularly primary outcomes.

**Conclusions:**

In conclusion, on the basis of WM, Shengmai injection or Shenmai injection may be a perforable treatment in CHF. In terms of insufficient of this study, more high quality RCTs needed to implement to support our conclusions.

**Electronic supplementary material:**

The online version of this article (10.1186/s12906-018-2090-3) contains supplementary material, which is available to authorized users.

## Background

Chronic heart failure (CHF) refers to a pathologic condition that cardiac output is absolute or relative reduce and cannot meet the whole body tissue metabolism under the normal venous return, then result in decreasing the myocardial contractile force and ventricular compliance, ultimately dyspnea, edema, feeble and so on. It was estimated that five-year survival rate of CHF was lower as malignant tumor and CHF was a main reason of disability and death on a global scale [1–3]. Impaired cardiac function of CHF patients may lessen their ability of daily living and render them a heavy economic pressure [1, 4]. At present, the primary aims of alleviating CHF symptoms are to inhibit myocardial remodeling, and perfect cardiac function [5]**.** Therefore, angiotensin-converting enzyme inhibitors (ACEIs), angiotensin II receptor blockers (ARBs), digoxin, and diuretics are become standard western medicine (WM) treatment in CHF [6], while it cannot obtain a desired effect own to poor compliance, lower heart rate of patients and others questions [5]. In consideration of its limitations, the application of Chinese herbal injections (CHIs) could be promoted. Currently, a combination between CHIs and WM treatment has already been a supportive measure in treatment of CHF in China. In accordance with traditional Chinese medicine (TCM) theories, CHF pertain to “heart impediment (xin bi)”, “palpitation”, “edema” and so forth, which caused by heart and then affect others organs. The clinical principle is to strengthen the body resistance to eliminate pathogenic factors [2]. Due to the relative low recognition of CHIs in CHF, this study selected six CHIs commonly used in CHF treatment, all of them were authorized by China Food and Drug Administration (CFDA), namely Huangqi injection (HQI), Shenfu injection (SFI), Shengmai injection (SI), Shenmai injection (SMI), Shenqi Fuzheng injection (SQFZI), Yiqifumai injection (YQFMI), to explore and rank their efficacy in CHF by the approach of network meta-analysis (NMA). Compared with conventional pairwise meta-analysis, NMA can sort the interventions via indirect comparison [7]**.** At the same time, the clinical trials compared those six CHIs head to head was lack. Thus, an attempt to conduct a NMA was necessarily. The goal of this study was to provide evidence-based hierarchies of the comparative efficacy and more insights for selection of CHF treatment.

## Methods

The study was congrunt with The Prisma Extension Statement for Reporting of Systematic Reviews Incorporating Network Meta-analyses of Health Care Interventions [8]. And the Prisma check list was displayed in Additional file 1.

### Eligibility criteria and study selection

A study was considered eligible if it suited for these criteria: 1) randomized controlled trial (RCT); 2) patients enrolled were diagnosed as CHF according to “Guidelines on the Diagnosis and Treatment of Heart Failure” conducted by The Chinese medical association cardiovascular epidemiology branch in 2014 [9] or “Clinical Guideline of New Drugs for Traditional Chinese Medicine” released by CFDA in 2002 [10]. Both of them contained both western diagnostics, the latter included TCM diagnostics as well; 3) patients receive WM treatment (e.g. cardiotonic, diuretic, ACEIs, β-blocker and so forth), meanwhile patients needed relevant therapy if they had complications during therapeutic process. On the basis of it, the treatment group received one of the included CHIs, the control group received another or just adopted WM. Besides, the dosages of CHIs were reported; 4) RCTs tested the clinical effective rate. The clinical effective rate calculated by this formula: (number of remarkable recovery patients + number of basic recovery patients) / total number of patients * 100%. Cardiac function classification was conformed to the standard issued by New York Heart Association (NYHA) in the United States. Clinical symptoms disappeared and cardiac function improved 2 levels at least was deemed as the class of remarkable recovery, clinical symptoms relieved and cardiac function increase 1 level was classified into the part of basic recovery, clinical symptoms and cardiac function was unaltered or worse belonged to deterioration. Besides, the incidence of left ventricular ejection fraction (LVEF), cardiac output (CO), stroke volume (SV), 6-min walk test (6MWT), brain natriuretic peptide (BNP), left ventricular end-diastolic dimension (LVEDD), left ventricular end- systolic dimension (LVESD), adverse drug reactions/adverse drug events (ADRs/ADEs) were also evaluated. The clinical effective rate and LVEF were regarded as dominating outcomes of the study, because the clinical effective rate can inflect the efficacy directly and LVEF was a main indicator for CHF. And others were counted as secondary outcomes. A study was excluded when it met these following criteria: 1) the study without full text; 2) duplicated reports; 3) RCTs with incomplete or inaccurate data; 4) RCTs with wrong sequence generation method. For example, sequence generated by odd or even date of birth, some rules based on date (or day) of admission and so forth; 5) patients received physiotherapy, acupuncture and moxibustion therapy, and Chinese materia medica preparation.

A comprehensive literatures searching was carried out in seven database including Embase, the Cochrane Library, Pubmed, Chinese Biological Medicine Database (CBM), China National Knowledge Infrastructure (CNKI), Wanfang Database, Chinese Scientific Journal Database (VIP) from their inception up to June 12nd 2017. In addition, there was no restriction on language. The method that incorporated the medical subject headings (MeSH) term and the free text was applied in searching process, and it would vary from different databases. Each searching item included three parts of terms that chronic heart failure, CHIs, and randomization. Detailed searching strategies were illustrated in Additional file 2.

After literatures duplicate checking, the rest literatures were firstly screened by titles and abstracts, reviews, irrelevant literatures and animals’ experiments reports were excluded. Literatures passed the initial filtration were read full text in order to sort out the eligible RCTs. Two reviewers undertook literature selection respectively, any divergences resolved by discussion or the third reviewer.

### Data extraction and quality assessment

Information from the eligible RCTs was extracted based on a custom-made form. The data consisted of the following items: 1) basic information of the eligibility: the first author, nationality, publication year, study desgin; 2) basic characteristics of patients: sample size, gender composition, average age, course of disease, primary diseases, cardiac function classification; 3) detail of RCTs’ intervention; 4) outcomes results and RCTs’ quality assessment.

The quality analysis was assessed with the Cochrane Collaboration’s tools (version 5.1.0 the Nordic Chchrane Center, the Cochrane Collaboration, 2012 Copenhagen, Denmark) by two reviewers independently. The tool comprised following these 7 items: 1) the method of randomization; 2) the concealment of random allocation; 3) the blinding method for patients and clinicians; 4) the blinding method for assessor; 5) the integrality of outcomes data; 6) the condition of selective reporting; 7) others bias. Each item was rated as “high risk”, “low risk” and “unclear”. And any difference between two reviewers settled by discussion or the third reviewer.

It is not necessary for this meta-analysis to obtain an ethical approval, because this study was the procedure that just gathered the clinical data in each RCT without any leak of patients’ information.

### Statistical analysis

NMA was performed with Stata software (version 12, Stata Corporation, College Station, Texas, U.S.) and Winbugs (version 1.4, MRC Biostatistics Unit, Cambridge, UK) software by using Mantel-Haenszel random-effects model. In Winbugs software, the number of iteration was set as 50,000, the first 20,000 was used for annealing algorithm in order to eliminate the impact of initial value. For binary outcomes, the pooled results were calculated as odds ratios (ORs). For continuous outcomes, mean differences (MD) were used. Both types of outcomes were presented with their 95% credible intervals (95% CIs) as well. Besides, the network graph showed indirect comparative relationship between different interventions was described. The node area of each intervention on behalf of its number of patients, and the thickness between different interventions represented the number of relative RCTs [11]. To rank various CHIs in treatment in CHF, the surface under the cumulative ranking curve (SUCRA) was utilized, which expressed each intervention’s efficacy with percentages. A larger area of SCUAR indicated that corresponding intervention was more preferable in certain outcomes [12]. After that, the funnel plots were depicted to reflect publication bias. Due to non-close loops in this NMA, the assumption of consistency between direct and indirect evidence was not utilized.

## Results

### Literature selection

**Fig. 1:**
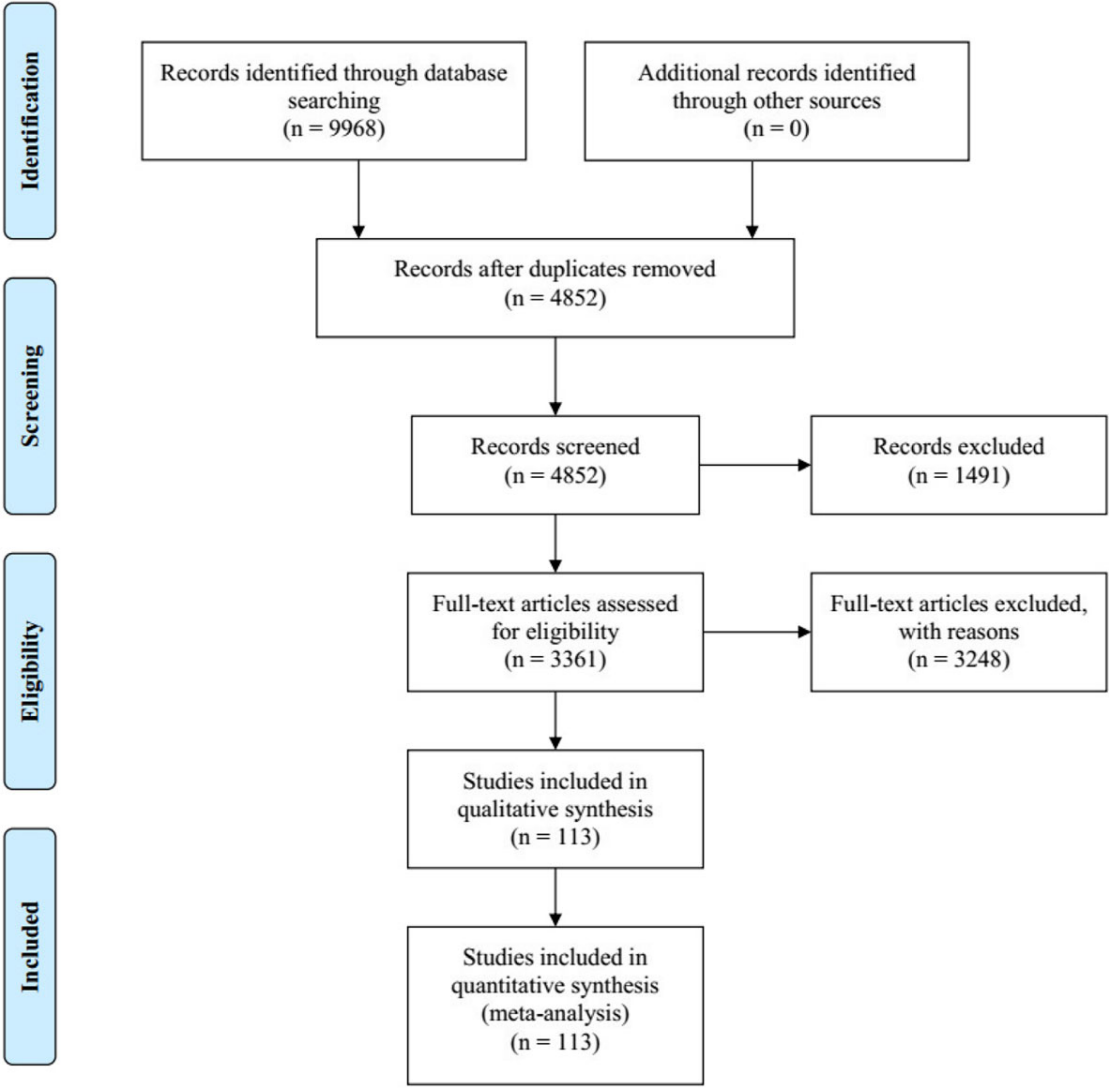
Flow chart of the search for eligible RCTs

A total of 9968 literatures were identified in initial search (Fig. 1). After removing duplicates, there were 4852 remained. By screened titles and abstracts, 1491 literatures were excluded because they were irrelevant literatures, reviews and animals’ experiments reports. 3361 literatures were eligible and then examined respectively, among which 3248 were further excluded, for the following reasons: 1) the RCT’s intervention or diseases missed eligibility criteria (*n* = 2694); 2) the therapeutic effect standard missed eligibility criteria (*n* = 256);3) the RCT with wrong randomization (*n* = 68); 4) the RCT did not divide patients in two groups (n = 68); 5) case reports (*n* = 40); 6) the RCT without full-text (*n* = 104); 7) the RCT with duplicated data (*n* = 18). As results, 113 RCTs that evaluated CHIs combined with WM for CHF were eligible in the NMA, and all of then carried out in China between 2001 and 2017. Meanwhile, 6 types of CHIs were identified, including HQI (12 RCTs), SFI (39 RCTs), SI (31 RCTs), SMI (13 RCTs), SQFZI (12 RCTs) and YQFMI (6 RCTs).

### Study characteristics and quality evaluation

**Fig. 2:**
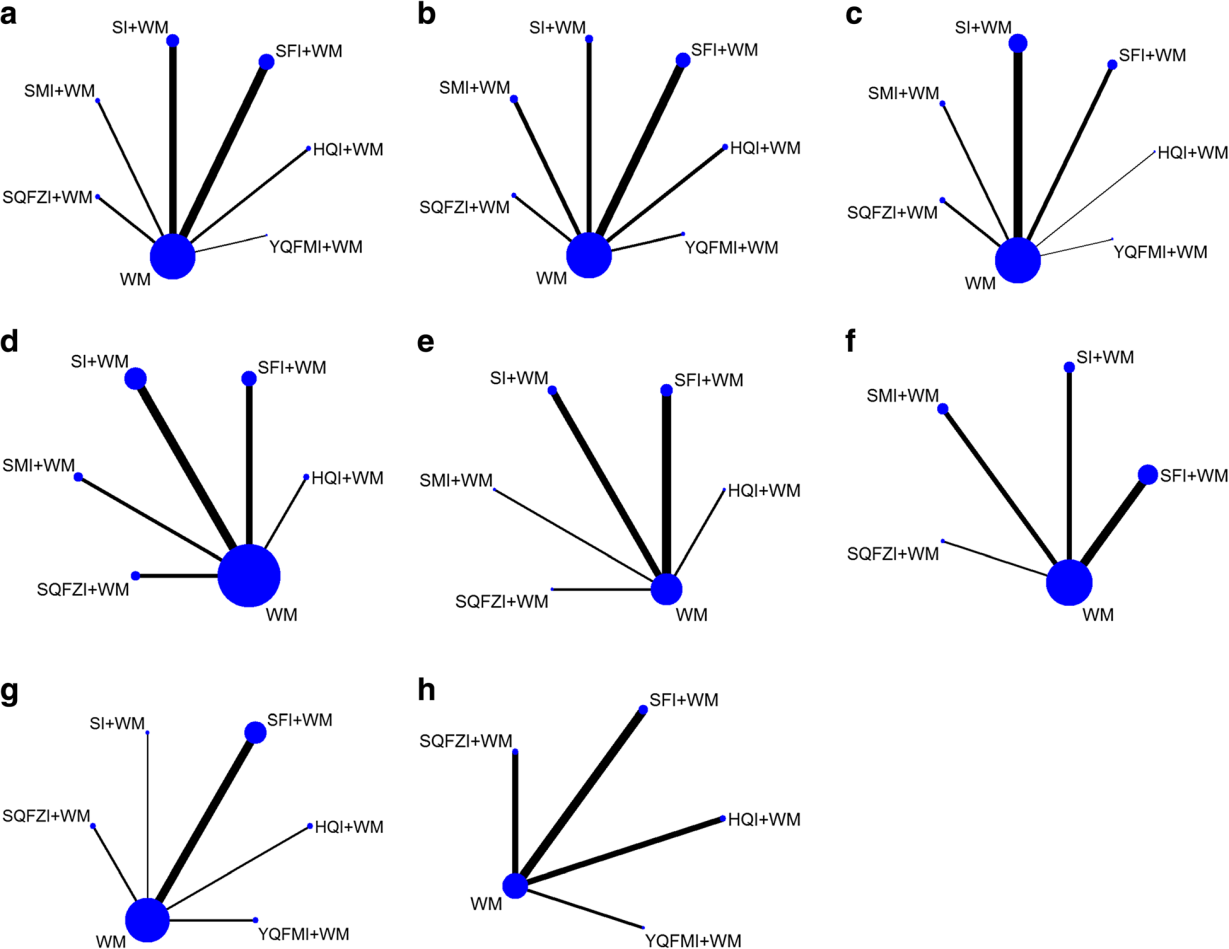
Network graph of the clinical effective rate, LVEF, CO, SV, 6MWT, BNP, LVEDD and LVESD. Note: **a**: the clinical effective rate; **b**: LVEF; **c**: CO; **d**: SV; **e**: 6MWT; **f**: BNP; **g**: LVEDD; **h**: LVESD

One hundered thirteen [3, 13–124] RCTs with 9525 patients were accorded with the eligible criteria, among which 4852 patients in the treatment groups and 4673 patients in the control groups. Among patients, the male patients were about 55% of total, and majority of patients were middle aged and elderly people. The intervention of the control groups were WM treatment, for instance, ACEIs, β-blocker, cardiotonic, diuretic. In the meantime, the treatment groups received one of the identified CHIs on the basis of the control groups. HQI, SFI, SI, SMI was a kind of injection that clinicians injected them with 5%–10% dextrose solution or 0.9% normal saline, the specific dosage were determined by clinicians. SQFZI was a kind of already made injection with menstruum. And YQFMI was a powder-injection, clinicians injected them with 5%–10% dextrose solution or 0.9% normal saline as well. All of identified CHIs were injected once a day via mainline. Characteristics of included RCTs can be found in Additional file 3. And the compared connections among each intervention for each outcome were displayed in Fig. 2.

**Fig. 3:**
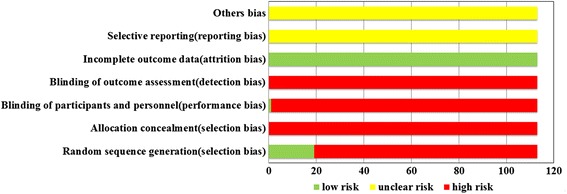
Risk of bias graph

For the eligible RCTs, 19 RCTs [15, 26, 32, 34, 37, 56, 57, 61, 64, 75, 81, 88, 96, 101, 103, 104, 107, 110, 118] used the random number table method or sortation randomization method to generate groups and 1 RCTs [88] utilized double blind method. Thus all of them were assessed as low risk. The rest RCTs were evaluated as high risk due to insufficient information. Besides, none of the included RCTs assessed had incomplete data, so the attrition bias was appraised as low risk. As for the part of reporting bias and others bias, the included RCTs did not provide relevant contents about selective report and mention any factors leading to high risk. Therefore these two items were evaluated as unclear risk. The graphical summary was depicted in Fig. 3.

### Outcomes

#### The clinical effective rate

The clinical effective rate was deemed as the primary outcomes, as shown in the right upper part of Table 1 [3, 13–124], HQI + WM (OR = 0.28, 95% CIs: 0.19–0.41),SFI + WM (OR = 0.29, 95% CIs: 0.24–0.35), SI + WM (OR = 0.28, 95% CIs: 0.22–0.35), SMI + WM (OR = 0.25, 95% CIs: 0.17–0.36), SQFZI+WM (OR = 0.28, 95% CIs: 0.19–0.39), YQFMI+WM (OR = 0.42, 95% CIs: 0.25–0.70), these six interventions with 95% CIs between 0 and 1 possessed the obvious strengthen in increasing clinical effective rate.

**Table 1:** Odds ratios/mean difference (95%CIs) of the clinical effective rate (right upper part) and LVEF (left lower part)

the clinical effective rate							
**LVEF**	**HQI + WM**	0.98(0.62,1.49)	1.03(0.62,1.59)	1.15(0.65,1.96)	1.00(0.60,1.76)	0.67(0.36,1.28)	0.28(0.19,0.41)
	0.13(−7.46,7.87)	**SFI + WM**	1.05(0.76,1.41)	1.17(0.79,1.83)	1.05(0.72,1.60)	0.69(0.42,1.21)	0.29(0.24,0.35)
	−4.31(−11.77,4.08)	− 4.50(−8.43,1.48)	**SI + WM**	1.12(0.73,1.75)	1.00(0.67,1.58)	0.66(0.39,1.16)	0.28(0.22,0.35)
	−3.08(−12.25,5.93)	−3.26(−9.21,3.26)	1.03(− 5.62,7.12)	**SMI + WM**	0.88(0.52,1.51)	0.59(0.31,1.10)	0.25(0.17,0.36)
	−2.60(−12.30,7.90)	− 2.69(− 10.87,5.50)	1.63(−6.79,9.48)	0.38(−8.25,9.34)	**SQFZI + WM**	0.66(0.36,1.23)	0.28(0.19,0.39)
	−3.11(−12.39,6.93)	− 3.17(− 10.26,4.48)	1.26(−6.88,8.28)	0.25(−8.18,8.53)	−0.50(− 10.09,9.22)	**YQFMI + WM**	0.42(0.25,0.70)
	4.27(− 2.61,11.46)	4.05(1.00,7.59)	8.61(4.22,10.99)	7.29(1.97,12.70)	6.81(−0.59,14.06)	7.26(0.42,13.64)	**WM**

Note: The result underlined meant it had statistical significant

**Fig. 4:**
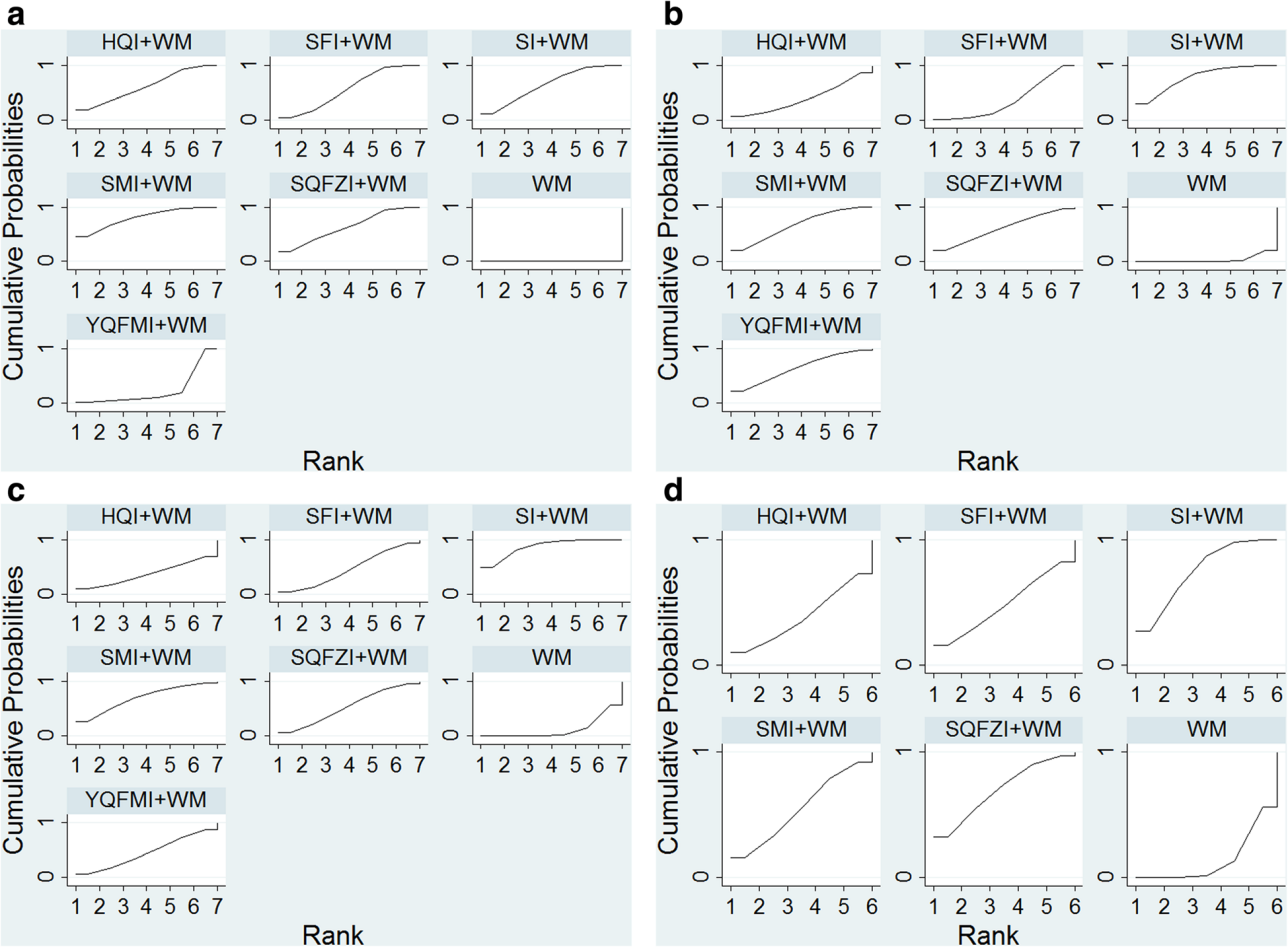
Plot of the surface under the cumulative ranking curves for all treatments on the clinical effective rate, LVEF, CO and SV. Note: **a**: the clinical effective rate; **b**: LVEF; **c**: CO; **d**: SV

In the Table 5 and Fig. 4, ranking analysis suggested that SMI + WM was the optimal combination, SI + WM was the second and the third was SQFZI+WM.

#### LVEF

As the other dominating outcomes, LVEF (%) was estimated in 57 RCTs [3, 13–15, 17, 19, 20, 23, 24, 32, 33, 36, 38, 39, 42, 45–49, 53, 55, 56, 58, 60, 61, 64, 65, 72, 75, 82, 84, 88, 91–93, 95–97, 99–103, 105, 106, 109–111, 114, 116, 117, 119–123]. According to Table 1, if the 95% CIs was more than 0, the result was significant. Four of them were noticeably better than WM treatment for LVEF, as SFI + WM (MD = 4.05, 95% CIs: 1.00–7.59), SI + WM (MD = 8.61, 95% CIs: 4.22–10.99), SMI + WM (MD = 7.29, 95% CIs: 1.97–12.70), YQFMI+WM (MD = 7.26, 95% CIs: 0.42–13.64) were outstanding among them compared with WM.

Results of ranking analysis manifested that SI + WM was efficacious in LVEF. Another beneficial treatments were SMI + WM and YQFMI+WM (Table 5 and Fig. 4).

#### Co

CO (L/min) was tested in 22 RCTs [20, 30, 31, 34, 45, 61, 68, 73, 75, 76, 81–83, 87, 92, 97, 99, 102, 111, 112, 117, 119] involved seven interventions. Based on Table 2, only SI + WM (MD = 1.29, 95% CIs: 0.74–1.72) had excellent performance in improving CO.

**Table 2:** Mean difference (95%CIs) of CO (right upper part) and SV (left lower part)

CO							
**SV**	**HQI + WM**	−0.22(−2.09,1.59)	−0.97(− 2.61,0.80)	−0.64(− 2.68,1.31)	−0.36(− 2.21,1.53)	−0.20(− 2.14,1.82)	0.32(− 1.29,1.99)
	−1.92(− 21.24,16.33)	**SFI + WM**	− 0.73(− 1.69,0.34)	−0.46(− 1.89,1.03)	−0.12(− 1.36,1.13)	0.03(−1.38,1.49)	0.55(− 0.29,1.43)
	− 6.07(− 21.13,9.09)	−4.32(− 17.07,9.69)	**SI + WM**	0.29(− 1.02,1.51)	0.61(− 0.46,1.58)	0.77(− 0.54,1.98)	1.29(0.74,1.72)
	−3.05(− 20.33,14.23)	−1.10(− 17.34,15.64)	2.99(− 8.76,14.46)	**SMI + WM**	0.32(− 1.15,1.77)	0.46(− 1.15,2.13)	0.99(− 0.17,2.16)
	− 5.74(− 23.58,11.31)	−3.84(− 20.16,12.65)	0.25(− 11.63,12.08)	− 2.51(− 17.27,11.40)	**SQFZI + WM**	0.14(− 1.31,1.65)	0.68(− 0.21,1.57)
	__	__	__	__	__	**YQFMI + WM**	0.52(− 0.64,1.68)
	3.28(− 10.90,17.25)	5.12(− 6.59,18.01)	9.35(3.75,14.90)	6.46(−3.94,16.34)	9.04(− 1.36,19.54)	__	**WM**

Note: The result underlined meant it had statistical significant

The SUCRA mentioned above was also affirmed, SI + WM was the best choice, and the following two were SMI + WM, and SQFZI+WM (Table 5 and Fig. 4).

#### SV

SV (ml) was reported in 20 RCTs [20, 23, 30, 36, 38, 45, 61, 68, 73, 75, 81, 82, 87, 92, 97, 99, 102, 111, 112, 117] involved six interventions. In terms of Table 2, only SI + WM (MD = 9.35, 95% CIs: 3.75–14.90) was remarkable among them.

Base on its SUCRA, SI + WM was the optimum, SQFZI+WM was the second and SMI + WM was the third (Table 5 and Fig. 4).

#### 6MWT

The potency of lengthening the distance of 6MWT (m) was assessed, and six interventions with 10 RCTs [24, 32, 39, 46, 51, 74, 85, 92, 106, 109] had data in contrast with WM, shown in Table 3. While the results showed no significant difference in most cases.

**Table 3:** Mean difference (95%CIs) of 6MWT (right upper part) and BNP (left lower part)

6MWT							
**BNP**	**HQI + WM**	− 5.40(− 71.99,70.70)	−29.10(− 115.00,66.32)	16.53(− 82.29,163.00)	7.97(−134.30,150.90)	__	17.38(− 52.09,84.04)
	__	**SFI + WM**	−22.05(− 91.18,37.25)	25.48(− 84.97,155.60)	15.06(− 137.40,161.20)	__	22.42(−9.55,53.97)
	__	54.65(−19.88,134.30)	**SI + WM**	50.83(− 75.61,178.60)	36.18(− 109.80,204.00)	__	45.03(− 12.06,101,10)
	__	80.17(16.67,147.5)	24.53(−58.64,110.20)	**SMI + WM**	−16.56(− 213.70,188.00)	__	− 1.23(− 133.50,100.10)
	__	110.00(35.08,186.40)	53.69(− 41.32,151.3)	27.54(− 44.17,108.30)	**SQFZI + WM**	__	__
	__	__	__	__	__	**YQFMI + WM**	7.12(−141.80,149.50)
	__	87.77(32.61,129.90)	34.25(−44.31,100.20)	8.33(−52.43,63.95)	−20.41(− 87.19,35.51)	__	**WM**

Note: The result underlined meant it had statistical significant

The ranking analysis indicated that SI + WM was the favorable intervention (Table 5).

#### BNP

In terms of BNP (pg/ml), five treatments with 21 RCTs [3, 33, 36, 38, 44, 46, 47, 53, 58, 64, 65, 69, 72, 88, 94–97, 104, 107, 109] were compared with WM in Table 3. SFI + WM vs SMI + WM (MD = 80.17, 95% CIs: 16.67–147.5), SFI + WM vs SQFZI+WM (MD = 110.00, 95% CIs: 35.08–186.40), SFI + WM vs WM (MD = 87.77, 95% CIs: 32.61–129.90) had statistically significance.

Based on ranking analysis, SQFZI+WM attained the first-rank (Table 5).

#### LVEDD & LVESD

The efficiency of decreasing LVEDD (mm) and LVESD (mm) was estimated as well. These two indexes were tested in 22 RCTs [13, 19, 20, 28, 30, 33, 36, 38, 39, 45, 53, 55, 58, 61, 65, 72, 107, 109, 111, 119, 121, 122] and 8 RCTs [13, 20, 30, 55, 61, 107, 109, 119] respectively. According to Table 4, it appeared that there was no significant difference between each comparison.

**Table 4:** Mean difference (95%CIs) of LVEDD (right upper part) and LVESD (left lower part)

LVEDD							
**LVESD**	**HQI + WM**	−6.39(− 20.58,8.88)	−5.90(− 22.48,11.36)	__	− 7.40(− 21.99,8.39)	−5.02(− 20.71,10.80)	− 7.87(− 21.64,7.22)
	−1.36(− 17.43,15.51)	**SFI + WM**	0.32(−9.47,10.49)	__	−1.04(− 6.88,5.10)	1.08(− 8.40,10.10)	−1.49(− 5.58,2.45)
	__	__	**SI + WM**	__	−1.43(− 11.31,8.84)	0.68(− 12.36,13.41)	−1.88(− 10.71,7.12)
	__	__	__	**SMI + WM**	__	__	__
	−2.21(−17.01,13.77)	−0.72(− 10.35,8.84)	__	__	**SQFZI + WM**	2.22(−7.61,11.31)	− 0.37(−5.41,3.66)
	0.97(− 20.42,23.54)	2.30(− 15.51,17.43)	__	__	3.02(− 14.80,20.13)	**YQFMI + WM**	−2.68(−10.91,6.07)
	− 4.03(− 18.02,10.96)	−2.57(− 10.73,5.24)	__	__	− 1.82(− 7.68,3.52)	−4.80(− 21.19,12.13)	**WM**

The ranking analysis suggested that HQI + WM and SQFZI+WM was the optimum for these two indexes (Table 5).

**Table 5:** Ranking probability for all treatments on the clinical effective rate, LVEF, CO, SV, 6MWT, BNP, LVEDD and LVESD

Treatment	Outcomes							
	the clinical effective rate	LVEF	CO	SV	6MWT	BNP	LVEDD	LVESD
HQI + WM	0.615	0.391	0.370	0.384	0.507	__	0.795	0.583
SFI + WM	0.559	0.359	0.462	0.481	0.596	0.020	0.503	0.533
SI + WM	0.649	0.783	0.872	0.747	0.776	0.392	0.499	__
SMI + WM	0.806	0.670	0.695	0.550	0.378	0.575	__	__
SQFZI+WM	0.635	0.613	0.530	0.697	0.460	0.849	0.361	0.501
YQFMI+WM	0.236	0.650	0.449	__	__	__	0.563	0.619
WM	0.00	0.034	0.122	0.141	0.284	0.664	0.279	0.263

#### ADRs/ADEs

Among 113 RCTs, a total of 36 [22, 23, 26, 30, 32, 33, 36, 39, 40, 43, 45, 48, 49, 51, 54, 64, 65, 67, 71–74, 77–81, 88, 93, 97–99, 103, 107, 108, 119, 120] RCTs (HQI (2 RCTs), SFI (13 RCTs), SI (13 RCTs), SMI (4 RCTs), SQFZI (2 RCTs), YQFMI (2 RCTs)) did not appear ADRs/ADEs during the trials. Another 72 RCTs (HQI (10 RCTs), SFI (25 RCTs), SI (17 RCTs), SMI (6 RCTs), SQFZI (10 RCTs), YQFMI (4 RCTs)) did not mention the situation of ADRs/ADEs. In others RCTs, one of the SFI treatment group occurred 2 cases of mild elevation of blood pressure and 2 cases of slight dry cough, and the corresponding control group occurred 3 cases of slight dry cough and 2 cases of headache [37]. Besides, one of the SI treatment group occurred 2 cases of mild anaphylaxis. There were 3 RCTs with SMI treatment group appeared ADRs [96, 100, 104]. One RCT occurred 1 case of pruritus in the treatment group and 6 cases mild headache in the control group. Another occurred 3 cases of mild gum bleeding in the control group. Another occurred 2 cases of stomach upset in the treatment and control group respectively. All of the symptom were alleviated after corresponding treatment and did not influence the RCTs.

#### Funnel plot characteristics

**Fig. 5:**
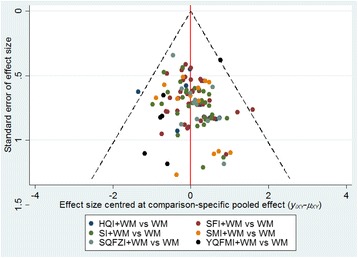
Funnel plot of the clinical effective rate

A comparison-adjusted funnel plot for the clinical effective rate was displayed in Fig. 5. The funnel plot was general symmetrical in visual. Thus we concluded that the obvious publication bias did not exist.

## Discussion

The impairment of CHF has been a global public health issue [125], with the utilization of a conjunction between CHIs and WM in its treatment, the efficacy of CHF has been promoted, meanwhile, more and more relevant RCTs and pairwise meta-analysis were carried out. But almost RCTs concerned about the efficacy between a kind of CHI plus WM and WM, many CHIs have not been compared head to head. Thus, researchers could merely figure out the efficacy of a CHI based on these RCTs via pairwise meta-analysis. While NMA can address this void, the efficacy of CHIs can be obtained at a time based on indirect comparison. By comparing with WM, the efficacy of CHIs for CHF and their rank can be demonstrated. we conducted a NMA in order to appraise the efficacy and safety of seven interventions: HQI + WM, SFI + WM, SI + WM, SMI + WM, SQFZI+WM, YQFMI+WM and WM.

This study made an extensive literature review and evaluation. The clinical data derived from 113 RCTs in the aspects of the clinical effective rate, LVEF, CO, SV, 6MWT, BNP, as well as the value of LVEDD and LVESD. CO, SV, LVEDD and LVESD was regarded as a supplement of cardiac condition, while the consequence of LVEDD and LVESD was no significant difference in most cases, these two outcomes’ results were merely deemed as a reference. Besides, 6MWT was vital indicator of patients’ recovery, and its importance was emphasized in the guide [9], though the amount of relevant RCTs in this study was small and its statistical power was low, we just treat it as a secondary outcome. In addition, the measurement of BNP was highlighted in guide as an exclusion for CHF [126]. Therefore, we viewed it as a secondary index as well. In terms of the primary outcomes, SI + WM and SMI + WM exhibited superior performance. What more, these two interventions did a noteworthy effect on CO and SV. And SI + WM also obtained a first-rank with respect to 6MWT. Overall, on the basis of receiving WM, CHF patients received SI or SMI may be more efficacious. Both of them were approved by CFDA on the market of CHF. SI was derived from Shengmai San which has been widely used for cardiovascular diseases since 1186 in China [127]. It was mainly made from the extractive of *Panax ginseng, Radix Ophiopogonis* and *Schisandra chinensis,* and had a function as replenishing qi-yin deficiency. Pharmacological researches have confirmed that SI had features in perfecting cardiac function and alleviating heart failure, enhancing myocardial contractility and cardiac pumping [128]. Under the guideline of TCM, SI was employed in CHF treatment routinely with its preferable curative effect, and several pairwise meta-analysis manifested that a conjunctive between SI and WM owned a superior capability on increasing the effective rate and LVEF [128–130]. As for SMI, it stemmed from Shenmai Yin which was prescribed by Simiao Sun in the Tang Dynasty [131], and its ingredients did not contain *Schisandra chinensis* compared with SI, but it also had a superior capacity in nourishing yin and benefiting qi. Upon pharmacological researches, the effect of SMI on promoting myocardial contractility and antiarrhythmic action has been verified [132]. Besides, several pairwise meta-analysis demonstrated that SMI plus WM exhibited a better performance in improving the effective rate, LVEF, CO, SV and decreasing BNP than WM [133–135].

Apart from efficacy, the safety of interventions was the other crucial element that must be considered in clinical trials. In this study, the occurrence rate of ADRs/ADEs was small, but about 64% of the research did not report the ADRs/ADEs. Hence, we could not draw a certain conclusion on it. As suggested in previous study, anaphylaxis was the main ADRs/ADEs of CHIs, and it would appear within 30 min at first time [136–139]. Hence, it is crucial for clinicians to monitor the ADRs/ADEs after using CHIs. Meanwhile, it is necessary to reported exactly if ADRs/ADEs occurred [136].

Upon the design and contents, three merits enhanced the creditable of this study. Firstly, this study made a comprehensive literature search and a contrast for six CHIs which have been already adopted in CHF treatment. Besides, this study expressed the efficacy of CHIs objectively due to the relevant large number of eligible RCTs. Furthermore, a strict eligibility criterion was formulated before implementing NMA. The consistency of the intervention and the curative standard lowered the clinical heterogeneity. What’s more, it was significant that the outcomes demonstrated cardiac condition in multiaspect. According to corresponding conclusions, this study provided several clinical suggestions for treatment in CHF.

### Limitation

Nevertheless, there was still insufficient in this study. Frist, the enrolled patients in RCTs were merely Chinese, which may lead to a bias on whether non-Chinese use eligible CHIs effectively or not. Although CHIs was mostly adopted in China, clinicians also can not only recruit Chinese. Next, just ten of included RCTs reported 6MWT in this study. While it is 6MWT, and readmission rate that associate with CHF patients closely and influence patients’ survival quality. Thus, these aspects should be paid more emphasis when RCTs are designed. In addition, the methodological quality was general, and most included RCTs did not mention the details of randomization and allocation concealment, which may generate an overestimate for eligible CHIs. It should be note that clinicians utilize low risk randomization and concealment method as possible. Based on the limitations, the RCTs conducted in the future should be perfected in relevant areas.

## Conclusion

To sum up, this study found that a combination between SI/SMI and WM exerted a positive effect on improving efficacy of CHF. However, the strength of evidence needed be promoted by more high quality RCTs. Moreover, safety of SI and SMI should be cautious monitoring in trials.

## Additional files

Additional file 1: PRISMA checklist for network meta-analysis. This file contained items about PRISMA checklist for network meta-analysis and corresponding pages of this study. (DOC 66 kb)

Additional file 2: Search strategy. This file contained the search strategy of traditional Chinese medicine injections and English database. (DOC 39 kb)

Additional file 3: Characteristics of included randomized controlled trials. This file contained the information about included randomized controlled trials. (DOC 38 kb)

## Abbreviations

6MWT: 6-min walk test
95% CIs: 95% credible intervals
ACEIs: Angiotensin-converting enzyme inhibitors
ADRs/ADEs: Dverse drug reactions/adverse drug events
ARBs: Angiotensin II receptor blockers
BNP: brain Natriuretic peptide
CBM: Chinese Biological Medicine Database
CFDA: China Food and Drug Administration
CHF: Chronic heart failure
CHIs: Chinese herbal injections
CNKI: China National Knowledge Infrastructure
CO: Cardiac output
HQI: Huangqi injection
LVEDD: Left ventricular end-diastolic dimension
LVEF: Left ventricular ejection fraction
LVESD: Left ventricular end-systolic dimension
MD: Mean differences
MeSH: Medical subject headings
NMA: Network meta-analysis
NYHA: New York Heart Association
ORs: Odds ratios
RCTs: Randomized controlled trials
SFI: Shenfu injection
SI: Shengmai injection
SMI: Shenmai injection
SQFZI: Shenqi Fuzheng injection
SUCRA: Surface under the cumulative ranking curve
SV: Stroke volume
TCM: Traditional Chinese medicine
VIP: Chinese Scientific Journal Database
WM: Western medicine
YQFMI: Yiqifumai injection
