# Supplementary material for: Comparative efficacy of Chinese herbal injections for treating chronic heart failure: a network meta-analysis
Source: BMC Complement Altern Med. 2018 Jan 31;18:41. doi: 10.1186/s12906-018-2090-3 (PMC5793420; doi:10.1186/s12906-018-2090-3)
Supplement: Supplementary file 2 — Search strategy. This file contained the search strategy of traditional Chinese medicine injections and English database. (DOC 39 kb) [file 12906_2018_2090_MOESM2_ESM.doc]

**Additional file 2. Search strategy**

**1. Search strategy of traditional Chinese medicine injections**

| Names of Chinese Herbal injections | English Searching Words | Chinese Searching Words |
| --- | --- | --- |
| Huangqi injection (HQI) | Huangqi OR Astragalus | 黄芪注射剂 OR 黄芪注射液 OR 注射液黄芪 |
| Shenfu injection (SFI) | Shenfu | 参附注射剂 OR 参附注射液 OR 注射液参附 |
| Shengmai injection (SI) | Shengmai | 生脉注射剂 OR 生脉注射液 OR 注射液生脉 |
| Shenmai injection (SMI) | Shenmai | 参麦注射剂 OR 参麦注射液 OR 注射液参麦 |
| Shenqi Fuzheng injection (SQFZI) | Shenqi Fuzheng | 参芪扶正注射剂 OR 参芪扶正注射液 OR 注射液参芪扶正 OR 参芪扶正 |
| Yiqifumai injection (YQFMI) | Yiqifumai | 益气复脉注射剂 OR 益气复脉注射液 OR 注射液益气复脉 |

**2. Search strategy of** **EMBASE**

#1 Heart Failure/exp

#2 Cardiac Failure

#3 Heart Decompensation

#4 Chronic heart failure

#5 Myocardial Failure

#6 Left -Sided Heart Failure

#7 Left Sided Heart Failure

#8 Right-Sided Heart Failure

#9 Right Sided Heart Failure

#10 Myocardial Failure

#11 Congestive Heart Failure

#12 Cardio-Renal Syndrome

#13 Paroxysmal Dyspnea

#14 Cardiac Edema

#15 #1 OR #2 OR #3 OR #4 OR #5 OR #6 OR #7 OR #8 OR #9 OR #10 OR #11 OR #12 OR #13 OR # 14

#16 Huangqi

#17 Astragalus

#18 Shengfu

#19 Shengmai

#20 Shenmai

#21 Shenqi Fuzheng

#22 Yiqifumai

#23 #16 OR #17 OR #18 OR #19 OR #20 OR #21 OR #22

#24 Randomized Controlled Trial/exp

#25 #15 AND #23 AND #24

**3. Search strategy of** **the Cochrane Library**

#1 Heart Failure: MeSH

#2 Paroxysmal Dyspnea: ti,ab,kw

#3 Cardiac Edema: ti,ab,kw

#4 Systolic Heart Failure: ti,ab,kw

#5 Diastolic Heart Failure: ti,ab,kw

#6 Cardio-Renal Syndrome: ti,ab,kw

#7 #1 OR #2 OR #3 OR #4 OR #5 OR #6

#8 Huangqi: ti,ab,kw

#9 Astragalus: ti,ab,kw

#10 Shengfu: ti,ab,kw

#11 Shengmai: ti,ab,kw

#12 Shenmai: ti,ab,kw

#13 Shenqi Fuzheng : ti,ab,kw

#14 Yiqifumai: ti,ab,kw

#15 #8 OR #9 OR #10 OR #11 OR #12 OR #13 OR #14

#16 random*

#17 #7 AND #15 AND #16

**4. Search strategy of** **Pubmed**

#1 Heart Failure [MeSH Terms]

#2 Cardiac Failure [Title/Abstract]

#3 Heart Decompensation [Title/Abstract]

#4 Chronic heart failure [Title/Abstract]

#5 Myocardial Failure [Title/Abstract]

#6 Left -Sided Heart Failure [Title/Abstract]

#7 Left Sided Heart Failure [Title/Abstract]

#8 Right-Sided Heart Failure [Title/Abstract]

#9 Right Sided Heart Failure [ Title/Abstract]

#10 Myocardial Failure [Title/Abstract]

#11 Congestive Heart Failure [Title/Abstract]

#12 Cardio-Renal Syndrome [Title/Abstract]

#13 Paroxysmal Dyspnea [Title/Abstract]

#14 Cardiac Edema [Title/Abstract]

#15 #1 OR #2 OR #3 OR #4 OR #5 OR #6 OR #7 OR #8 OR #9 OR #10 OR #11 OR #12 OR #13 OR # 14

#16 Huangqi [Title/Abstract]

#17 Astragalus [Title/Abstract]

#18 Shengfu [Title/Abstract]

#19 Shengmai [Title/Abstract]

#20 Shenmai [Title/Abstract]

#21 Shenqi Fuzheng [Title/Abstract]

#22 Yiqifumai [Title/Abstract]

#23 #16 OR #17 OR #18 OR #19 OR #20 OR #21 OR #22

#24 Randomized Controlled Trial [Publication Type]

#25 Controlled Clinical Trial [Publication Type]

#26 random* [All Fields]

#27 #24 OR #25 OR #26

#28 #15 AND #23 AND #27
