# Supplementary material for: Comparative efficacy of Chinese herbal injections for treating chronic heart failure: a network meta-analysis
Source: BMC Complement Altern Med. 2018 Jan 31;18:41. doi: 10.1186/s12906-018-2090-3 (PMC5793420; doi:10.1186/s12906-018-2090-3)
Supplement: Supplementary file 3 — Characteristics of included randomized controlled trials. This file contained the information about included randomized controlled trials. (DOC 38 kb) [file 12906_2018_2090_MOESM3_ESM.doc]

**Additional file 3. Characteristics of included randomized controlled trials**

**Abbreviation: T: treatment group; C: control group; M: male; F: female; HQI: Huangqi injection; SFI: Shenfu injection; SI: Shengmai injection; SMI: Shenmai injection; SQFZI: Shenqi Fuzheng injection; YQFMI: Yiqifumai injection; WM: western medicine. Outcomes: 1: the clinical effective rate; 2: left ventricular ejection fraction; 3: cardiac output; 4: stroke volume; 5: 6-minute walk test; 6: brain natriuretic peptide; 7: left ventricular end-diastolic dimension; 8: left ventricular end- systolic dimension; 9: adverse drug reactions/adverse drug events.**

| Studies | Sample size(T/C) | Sex  (M/F) | Average age | Course of disease (year) | Primary diseases | Cardiac function classification (T/C) | | | Treatment group | Control group | Course of treatment (day) | Outcomes |
| --- | --- | --- | --- | --- | --- | --- | --- | --- | --- | --- | --- | --- |
|  |  |  |  |  |  | II | III | IV |  |  |  |  |
| Yuan 2003 | 30/30 | 17/43 | 49-78 | 3-21 | coronary disease: 26 cases, hypertensive heart disease: 14 cases, rheumatic heart disease: 10 cases, dilated cardiomyopathy: 6 cases, pulmonary heart disease: 4 cases | 8 | 35 | 17 | HQI 40ml+WM | WM | 14 | 1,9 |
| Luo 2003 | 30/30 | 25/35 | T: 52±4.5 C: 52±3.5 | T: 5±2.1 C: 5±1.9 | coronary disease: 28 cases, hypertensive heart disease: 9 cases, rheumatic heart disease: 9 cases, pulmonary heart disease: 7 cases, viral myocarditis: 7 cases | 2/4 | 19/18 | 9/8 | HQI 40ml+WM | WM | 14 | 1 |
| Gu 2003 | 68/66 | 64/70 | 55.8 | 0.6-10 | … | 49 | 45 | 40 | HQI 40ml+WM | WM | 28 | 1,2 |
| Gao 2005 | 40/36 | 37/39 | 70±8 | 0.5-10 | … | 35 | 31 | 10 | HQI 40ml+WM | WM | 30 | 1 |
| Xing 2006 | 22/21 | 13/30 | 60.0±15.6 | 6.2±4.1 | ischemic heart disease: 23 cases, dilated cardiomyopathy: 20 cases | 15 | 16 | 12 | HQI 50ml+WM | WM | 14 | 1,2,7,8 |
| Feng 2008 | 34/32 | 33/33 | T: 57.83±7.79 C: 57.93±7.71 | T: 5.27±4.00 C: 5.07±3.80 | … | 16/14 | 13/15 | 5/3 | HQI 40ml+WM | WM | 28 | 1,2 |
| Yan 2009 | 80/79 | 64/95 | T: 58.1±11.9 C: 57.8±10.2 | … | coronary disease: 70 cases, hypertensive heart disease: 32 cases, rheumatic heart disease: 21 cases, dilated cardiomyopathy: 17 cases, pulmonary heart disease: 19 cases | … | | | HQI 50ml+WM | WM | 15 | 1,2,7 |
| Lin 2009 | 31/30 | 13/48 | T: 68.8±9.7 C: 69.5±9.2 | … | coronary disease: 36 cases, , dilated cardiomyopathy: 7 cases, pulmonary heart disease: 18 cases | 9/8 | 10/11 | 12/11 | HQI 10ml+WM | WM | 14 | 1,2,3,4,7,8 |
| Wang 2013 | 31/29 | 21/39 | T: 66.8 C: 67.6 | … | … | / | 42 | 18 | HQI 40ml+WM | WM | 15 | 1,2,4,9 |
| Zhang 2013 | 29/28 | 23/34 | T: 62.1±5.1 C: 61.8±4.5 | … | … | … | | | HQI 40ml+WM | WM | 14 | 1 |
| Jia 2013 | 49/49 | 54/44 | T: 66.46±9.15 C: 67.55±8.58 | T: 2.25±0.48 C: 2.19±1.02 | … | 29/27 | 20/22 | / | HQI 30ml+WM | WM | 14 | 1,2,5 |
| Zhang 2017 | 45/45 | 52/38 | T: 56.72±7.45 C: 58.19±7.86 | … | coronary disease: 37 cases, hypertensive heart disease: 31 cases, rheumatic heart disease: 8 cases, dilated cardiomyopathy: 14 cases | 15/14 | 17/18 | 13/13 | HQI 20ml+WM | WM | … | 1,2 |
| Liu 2005 | 48/49 | 56/41 | T: 63±19 C: 62±19 | T: 14±10 C: 17±11 | ischemic heart disease: 40 cases, hypertensive heart disease: 35 cases, rheumatic heart disease: 13cases, dilated cardiomyopathy: 9 cases, | 14/12 | 16/17 | 18/20 | SFI 40-80ml+WM | WM | 21 | 1,2,3,4,7,8 |
| Liu 2008 | 40/40 | 48/32 | 56-82 | … | coronary disease: 58 cases, hypertensive heart disease: 8 cases, rheumatic heart disease: 2 cases, dilated cardiomyopathy: 1 cases, pulmonary heart disease: 11 cases | 8/10 | 25/24 | 7/6 | SFI 40ml+WM | WM | 21 | 1,9 |
| Wu 2008 | 31/32 | 24/39 | T: 72.94±7.58 C: 76.43±6.80 | T: 8.90±6.40 C: 9.69±4.88 | … | / | 20/22 | 11/10 | SFI 40ml+WM | WM | 7 | 1,3,4,7,8,9 |
| Wu 2009 | 33/29 | 31/31 | T: 71.48±5.78 C: 73.59±6.96 | … | … | / | 6/5 | 27/24 | SFI 50ml+WM | WM | 14 | 1,2,4,6,7,9 |
| Zhang 2009 | 23/22 | 26/19 | 74 | T: 0.6-15 C: 0.5-12 | … | … | | | SFI 40ml+WM | WM | 14 | 1,2 |
| Yang 2010 | 40/40 | 42/38 | T: 70.1 C: 69.5 | T: 5.1 C: 4.9 | coronary disease: 37 cases, hypertensive heart disease: 25 cases, rheumatic heart disease: 18 cases | … | | | SFI 50ml+WM | WM | 15 | 1,2,5,7,9 |
| Yao 2010 | 30/30 | 33/27 | 53.6±9.8 | … | coronary disease: 36 cases, hypertensive heart disease: 16 cases, dilated cardiomyopathy: 8 cases | 16 | 34 | 10 | SFI 50ml+WM | WM | 14 | 1,2,3,4,7,9 |
| Qiu 2010 | 85/85 | 81/89 | T: 62.80±13.10 C: 61.40±12.40 | … | coronary disease: 84 cases, hypertensive heart disease: 23 cases, rheumatic heart disease: 17 cases, dilated cardiomyopathy: 10 cases, pulmonary heart disease: 36 cases | 24/25 | 26/27 | 35/33 | SFI 50ml+WM | WM | 14 | 1 |
| Bao 2011 | 30/30 | 32/28 | 56.3±9.8 | … | coronary disease: 40 cases, hypertensive heart disease: 15 cases, dilated cardiomyopathy: 5 cases | 14 | 40 | 6 | SFI 50ml+WM | WM | 14 | 1 |
| Cao 2011 | 44/43 | 50/37 | T: 69.20±8.62 C: 69.70±8.54 | … | coronary disease: 47 cases, hypertensive heart disease: 32 cases, rheumatic heart disease: 8 cases | 12/11 | 24/23 | 8/9 | SFI 50ml+WM | WM | 15 | 1,9 |
| Jiang 2011 | 96/96 | … | … | … | … | … | | | SFI 60ml+WM | WM | 14 | 1 |
| Fu 2011 | 50/46 | 59/37 | T: 54±10 C: 55±11 | T: 2-11 C: 2.5-10 | coronary disease: 45 cases, hypertensive heart disease: 22 cases, rheumatic heart disease: 20 cases, dilated cardiomyopathy: 9 cases | 18/16 | 25/22 | 7/8 | SFI 60ml+WM | WM | 60 | 1,2,7,8 |
| Liu 2011 | 71/69 | 82/58 | T: 45.04±2.61 C: 45.31±3.02 | … | … | 40/41 | 31/28 |  | SFI 40ml+WM | WM | 14 | 1,5,9 |
| Zhang 2011 | 40/40 | 45/35 | T: 62.00±6.90 C: 61.00±7.20 | … | coronary disease: 42 cases, hypertensive heart disease: 21 cases, pulmonary heart disease: 17 cases | 9/10 | 23/23 | 8/7 | SFI 30ml+WM | WM | 20 | 1,2, |
| Gao 2012 | 30/30 | 31/29 | T: 61.00±7.70 C: 59.33±7.27 | T: 10.81±2.49 C: 9.82±2.28 | … | … | | | SFI 60ml+WM | WM | 14 | 1 |
| Li 2012 | 35/35 | 40/30 | T: 68.4 C: 67.7 | … | coronary disease: 22 cases, dilated cardiomyopathy: 7 cases, pulmonary heart disease: 41 cases | 8/6 | 27/29 | / | SFI 60ml+WM | WM | 15 | 1,9 |
| Fan 2012 | 40/40 | 43/37 | 65-89 | … | … | / | 33/31 | 7/9 | SFI 100ml+WM | WM | 14 | 1,9 |
| Ran 2012 | 49/47 | 48/48 | T: 61.8±12.3 C: 62.4±10.7 | T: 11.8±6.4 C: 12.6±7.1 | coronary disease: 42 cases, hypertensive heart disease: 18 cases, rheumatic heart disease: 12 cases, dilated cardiomyopathy: 9 cases, pulmonary heart disease: 15 cases | 10/8 | 24/26 | 15/13 | SFI 60ml+WM | WM | 14 | 1,2,5,6 |
| Wang 2012 | 51/49 | 58/42 | T: 45.05±2.61 C: 45.61±3.02 | … | … | 29/29 | 22/20 | / | SFI 40ml+WM | WM | 14 | 1 |
| Zhou 2013 | 30/30 | 34/26 | T: 77.21 C: 76.32 | T: 9.63 C: 9.24 | … | 5/6 | 17/18 | 8/6 | SFI 40ml+WM | WM | 14 | 1 |
| Qiu 2013 | 40/40 | 35/45 | T: 79.00±8.27 C: 77.00±11.57 | … | coronary disease: 73 cases, dilated cardiomyopathy: 7 cases | / | 34/35 | 6/5 | SFI 50ml+WM | WM | 10 | 1,2,9 |
| Ma 2013 | 48/50 | … | … | … | … | … | | | SFI 50ml+WM | WM | 14 | 1,6 |
| Wu 2013 | 30/30 | 49/11 | T: 70.4±2.4 C: 68.9±3.1 | T: 9.4±1.2 C: 9.3±2.1 | … | / | 22/24 | 8/6 | SFI 50ml+WM | WM | 14 | 1,2,6 |
| Ding 2013 | 40/40 | 41/39 | T: 72.94±7.58 C: 76.43±6.80 | T: 8.90±6.40 C: 9.69±4.88 | … | / | 29/30 | 11/10 | SFI 60ml+WM | WM | 14 | 1,2,6 |
| Zhu 2013 | 30/30 | 26/34 | T: 64.54±6.01 C: 66.47±6.45 | T: 10.52±5.46 C: 9.87±6.43 | coronary disease: 28 cases, hypertensive heart disease: 27 cases, atrial fibrillation: 5 cases | … | | | SFI 50ml+WM | WM | 10 | 1,2,9 |
| Chen 2014 | 36/32 | 41/27 | T: 58.32±6.97 C: 57.96±7.20 | T: 7.96±5.32 C: 8.38±4.28 | coronary disease: 36 cases, hypertensive heart disease: 7 cases, rheumatic heart disease: 6 cases, dilated cardiomyopathy: 6 cases, pulmonary heart disease: 13 cases | 8/6 | 16/15 | 12/11 | SFI 100ml+WM | WM | 14 | 1,3 |
| Cheng 2014 | 88/72 | 100/60 | T: 56.7±11.6 C: 58.4±9.6 | … | ischemic heart disease with coronary diaease: 77 cases, hypertensive heart disease: 21 cases, dilated cardiomyopathy: 32 cases, pulmonary heart disease: 30 cases | 28/23 | 48/40 | 12/9 | SFI 50ml+WM | WM | 14 | 1,2,6,7,9 |
| Lv 2014 | 58/55 | 61/52 | T: 69.4±2.1 C: 67.2±2.6 | T: 4.9±2.1 C: 5.7±1.4 | coronary disease: 56 cases, hypertensive heart disease: 31 cases, dilated cardiomyopathy: 9 cases, coronary disease with hypertension: 17 cases | 19/15 | 28/29 | 11/11 | SFI 50ml+WM | WM | 15 | 1,2,4,6,7 |
| Wang 2014 | 50/50 | 60/40 | T: 58.32±6.97 C: 58.21±6.30 | T: 7.96±5.32 C: 9.31±4.15 | coronary disease: 48 cases, hypertensive heart disease: 11 cases, rheumatic heart disease: 6 cases, dilated cardiomyopathy: 13 cases, pulmonary heart disease: 22 cases | 12/16 | 22/25 | 9/16 | SFI 50ml+WM | WM | 14 | 1 |
| Yu 2014 | 72/75 | 83/64 | 35-80 | … | coronary disease: 62 cases, hypertensive heart disease: 38 cases, dilated cardiomyopathy: 18 cases, coronary disease with hypertension: 29 cases | … | | | SFI 50ml+WM | WM | 14 | 1 |
| Jin 2015 | 40/40 | 46/34 | T: 62.02±4.30 C: 62.11±4.11 | T: 7.20±1.22 C: 7.43±1.28 | coronary disease: 57 cases, rheumatic heart disease: 14 cases, dilated cardiomyopathy: 9 cases | … | | | SFI 50ml+WM | WM | 60 | 1,2 |
| Ren 2015 | 30/30 | 37/23 | T: 71.3±6.8 C: 70.9±6.3 | T: 6.8±3.5 C: 6.5±3.6 | ischemic heart disease: 33 cases, hypertensive heart disease: 17 cases, rheumatic heart disease: 5cases, dilated cardiomyopathy: 2 cases, pulmonary heart disease: 3 cases | 5/6 | 21/21 | 4/3 | SFI 50ml+WM | WM | 14 | 1,2,5,9 |
| Qian 2015 | 30/30 | 27/33 | 65-80 | … | … | / | 21/18 | 9/12 | SFI 50ml+WM | WM | 14 | 1,2,6,7 |
| Wang 2015 | 45/45 | 59/31 | T: 69.2±10.4 C: 68.7±9.7 | T: 4.60±2.60 C: 4.36±2.70 | coronary disease: 48 cases, hypertensive heart disease: 28 cases, cardiomyopathy: 14 cases | 11/10 | 24/25 | 10/10 | SFI 20ml+WM | WM | 14 | 1,2,6,7 |
| Xu 2015 | 25/25 | 32/18 | T: 63.00±10.00 C: 64.00±11.00 | T: 6.4 C: 5.6 | coronary disease: 20 cases, hypertensive heart disease: 19 cases, rheumatic heart disease: 2 cases, dilated cardiomyopathy: 9 cases | 4/5 | 11/11 | 10/9 | SFI 100ml+WM | WM | 7 | 1,7 |
| Li 2016 | 20/20 | 24/16 | T: 64.53 C: 60.45 | T: 9.65 C: 9.97 | … | … | | | SFI 50ml+WM | WM | 10 | 1,3 |
| Wang 2016 | 26/30 | 29/27 | T: 71.56±2.47 C: 70.23±1.56 | T: 16.42±14.58 C: 17.35±15.74 | … | … | | | SFI 60ml+WM | WM | 10±2 | 1,9 |
| Cui 2016 | 30/30 | 33/27 | T: 68.6±11.1 C: 68.5±9.2 | T: 6.3±3.3 C: 5.92±3.2 | coronary disease: 34 cases, hypertensive heart disease: 7 cases, dilated cardiomyopathy: 19 cases | 5/6 | 10/8 | 15/16 | SFI 100ml+WM | WM | 14 | 1 |
| Mao 2016 | 100/100 | 116/84 | 56.2 | … | … | … | | | SFI 40ml+WM | WM | 5-10 | 1, |
| Tang 2001 | 33/33 | 41/25 | T: 65.3 C: 64.7 | T: 3-12 C: 2-10 | coronary disease: 23 cases, hypertensive heart disease: 7 cases, rheumatic heart disease: 19 cases, dilated cardiomyopathy: 4 cases, pulmonary heart disease: 13 cases | … | | | SI 20ml+WM | WM | 14 | 1,9 |
| Li 2002 | 28/25 | 32/21 | T: 67.6 C: 66.4 | T: 2-22 C: 3-20 | coronary disease: 21 cases, hypertensive heart disease: 15 cases, rheumatic heart disease: 7 cases, dilated cardiomyopathy: 3 cases, pulmonary heart disease: 7 cases | / | 15/14 | 13/11 | SI 20-40ml+WM | WM | 15 | 1,9 |
| Xu 2004 | 50/26 | 47/29 | T: 62 C: 62.5 | T: 5.6 C: 5.8 | … | / | 32/17 | 18/9 | SI 100ml+WM | WM | 15 | 1,3,4,9 |
| Kong 2004 | 30/20 | 29/21 | T: 48-70 C: 46-70 | T: 4-30 C: 4-28 | … | 9/7 | 15/10 | 6/3 | SI 20-40ml+WM | WM | 14 | 1,3 |
| Wang 2004 | 31/31 | 44/18 | T: 60.5±7.3 C: 59.5±6.4 | T: 7.8±4.2 C: 7.5±4.5 | coronary disease: 18 cases, hypertensive heart disease: 17 cases, rheumatic heart disease: 9 cases, dilated cardiomyopathy: 9 cases, pulmonary heart disease: 9 cases | 8/10 | 13/12 | 10/9 | SI 20-30ml+WM | WM | 14 | 1,9 |
| He 2006 | 20/15 | 22/13 | 59.5 | T: 8.2 C: 8 | … | 5/2 | 8/8 | 7/5 | SI 40ml+WM | WM | 20 | 1,2,3,4 |
| Cheng 2007 | 45/45 | 46/44 | T: 65.45 C: 64.32 | … | coronary disease: 44 cases, hypertensive heart disease: 27 cases,dilated cardiomyopathy: 19 cases | 10/12 | 22/23 | 13/10 | SI 40-60ml+WM | WM | 14 | 1,9 |
| Ni 2007 | 28/22 | 29/21 | T: 56.5±7.5 C: 46.8±5.2 | T: 9.8±4.8 C: 8.6±5.4 | coronary disease: 17 cases, hypertensive heart disease: 7 cases, rheumatic heart disease: 20 cases, pulmonary heart disease: 6 cases | 13/10 | 15/12 | / | SI 100-200ml+WM | WM | 14 | 1 |
| Wang 2007 | 80/80 | 85/75 | T: 42-86 C: 41-88 | … | … | 14/15 | 38/36 | 28/29 | SI 40-60mll+WM | WM | 14 | 1 |
| Chen 2008 | 36/34 | … | 64.28 | 0.5-8 | coronary disease: 24 cases, rheumatic heart disease: 4 cases, dilated cardiomyopathy: 8 cases, pulmonary heart disease: 9 cases, chronic obstructive pulmonary disease: 1 case, coronary disease with hypertension: 22 cases, others: 2 cases | 18 | 42 | 10 | SI 1ml/(kg· d), the maximum dose: 60ml/d | WM | 14 | 1,3 |
| Liu 2008 | 60/60 | 84/36 | 64 | 1-12 | coronary disease: 64 cases, hypertensive heart disease: 10 cases, rheumatic heart disease: 12 cases, dilated cardiomyopathy: 28 cases, degenerative valvular heart disease: 6 cases | … | | | SI 1ml/(kg· d), the maximum dose: 60ml/d | WM | 14 | 1 |
| Zhou 2009 | 32/32 | … | … | … | … | … | | | SI 40-60ml+WM | WM | 28 | 1,2,6,7,9 |
| Zhai 2009 | 30/30 | 34/26 | T: 62±6 C: 63±6 | T: 10±4 C: 10±5 | coronary disease: 39 cases, hypertensive heart disease: 6 cases, rheumatic heart disease: 7 cases, dilated cardiomyopathy: 4 cases, pulmonary heart disease: 2 cases, congenital heart disease: 2 cases | / | 17/18 | 13/12 | SI 40ml+WM | WM | 15 | 1,3,4,9 |
| Li 2009 | 30/30 | 34/26 | … | 0.2-20 | coronary disease: 19 cases, hypertensive heart disease: 21 cases, rheumatic heart disease: 5 cases, dilated cardiomyopathy: 4 cases, pulmonary heart disease: 4 cases, senile degenerative valvular heart disease: 3 cases, congenital heart disease: 2 cases, syphilitic heart disease: 1 case, hyperthyroid heart disease: 1 case | 6 | 36 | 18 | SI 50ml+WM | WM | 14 | 1,2 |
| Shi 2009 | 50/30 | … | … | … | … | … | | | SI 40ml+WM | WM | 14 | 1,2,9 |
| Kong 2010 | 50/50 | 58/42 | 67.4±9.1 | … | coronary disease: 42 cases, hypertensive heart disease: 30 cases, rheumatic heart disease: 8 cases, cardiomyopathy: 2 cases, pulmonary heart disease: 15 cases, congenital heart disease: 3 cases | 6/7 | 25/27 | 19/16 | SI 40ml+WM | WM | 7 | 1,2,6,9 |
| Wang 2010 | 38/36 | 43/31 | T: 71.5±9.4 C: 72.3±8.6 | … | coronary disease: 41 cases, hypertensive heart disease: 29 cases, dilated cardiomyopathy: 4 cases | 4/3 | 22/23 | 12/10 | SI 20ml+WM | WM | 15 | 1,2,6,9 |
| Yang 2010 | 43/40 | 45/38 | T: 55.4±11.8 C: 56.3±8.9 | … | coronary disease: 31 cases, hypertensive heart disease: 25 cases, dilated cardiomyopathy: 21 cases, congenital heart disease: 4 cases, valvular heart disease: 2 cases | 12/11 | 17/16 | 14/13 | SI 60ml+WM | WM | 28 | 1 |
| Wang 2010 | 41/41 | 50/32 | T: 63.6±7.2 C: 64.3±7.7 | T: 5.8±2.5 C: 6.2±2.3 | coronary disease: 63 cases, rheumatic heart disease: 10 cases, dilated cardiomyopathy: 9 cases | … | | | SI 40ml+WM | WM | 14 | 1 |
| Zou 2011 | 30/30 | 40/20 | T: 67.35±5.6 C: 64.27±4.98 | T: 4.52±1.86 C: 4.88±1.43 | coronary disease: 38 cases, hypertensive heart disease: 8 cases, pulmonary heart disease: 14 cases | 7/8 | 14/12 | 9/10 | SI 40ml+WM | WM | 14 | 1,3,4 |
| Wu 2011 | 36/34 | 36/34 | T: 67.53±10.29 C: 62.47±11.32 | … | … | … | | | SI 50ml+WM | WM | 14 | 1,6 |
| Lu 2012 | 68/68 | 89/47 | T: 61.30±5.81 C: 60.72±6.20 | … | coronary disease: 95 cases, rheumatic heart disease: 22 cases, dilated cardiomyopathy: 19 cases | 20/28 | 48/50 | | SI 50ml+WM | WM | 14 | 1,5 |
| Ni 2012 | 43/41 | … | … | … | … | … | | | SI 50ml+WM | WM | 14 | 1,2,6,7,9 |
| Wu 2013 | 30/22 | 33/19 | T: 43-81 C: 45-78 | T: 5-16 C: 4-12 | … | 10/7 | 17/13 | 3/2 | SI 60ml+WM | WM | 14 | 1,2 |
| Wen 2013 | 32/32 | 35/29 | T: 61±5 C: 64±8 | T: 2-10 C: 2-16 | … | / | 18/17 | 14/15 | SI 50ml+WM | WM | 14 | 1,2,3,4,9 |
| Pan 2014 | 21/21 | 29/13 | 41-76 | … | coronary disease: 26 cases, hypertensive heart disease: 11 cases, rheumatic heart disease: 3 cases, dilated cardiomyopathy: 2 cases | … | | | SI 50ml+WM | WM | 14 | 1 |
| Luo 2014 | 38/38 | 51/25 | T: 75.4±6.80 C: 73.7±7.18 | T: 3-12 C: 2-14 | coronary disease: 44 cases, hypertensive heart disease: 12 cases, rheumatic heart disease: 13 cases, dilated cardiomyopathy: 7 cases | 12/13 | 23/21 | 3/4 | SI 40ml+WM | WM | 14 | 1,9 |
| Zhang 2015 | 53/50 | 57/46 | 63±6 | 7±3 | coronary disease: 39 cases, hypertensive heart disease: 28 cases, rheumatic heart disease: 9 cases, dilated cardiomyopathy: 5 cases, pulmonary heart disease: 22 cases | … | | | SI 60ml+WM | WM | 15 | 1,2,3,4,5 |
| Zhao 2015 | 31/31 | 30/32 | T: 65.77±4.46  C: 67.61±4.28 | T: 2-14 C: 2-11 | … | 6/5 | 13/12 | 12/14 | SI 60ml+WM | WM | 14 | 1,3,4 |
| Chen 2016 | 43/43 | 52/34 | T: 65.8±7.1 C: 65.2±6.8 | T: 1-11 C: 1-9 | coronary disease: 45 cases, hypertensive heart disease: 21 cases, rheumatic heart disease: 9 cases, dilated cardiomyopathy: 11 cases | 12/11 | 25/27 | 6/5 | SI 40ml+WM | WM | 14 | 1,9 |
| Wen 2016 | 20/20 | 23/17 | T: 64.75±3.85 C: 64.35±3.75 | T: 1-20 C: 1-20 | … | 7/6 | 11/12 | 2/2 | SI 40-60ml+WM | WM | 14 | 1,5,9 |
| Wang 2002 | 22/18 | 25/15 | T: 38-75 C: 39-76 | T: 0.6-8 C: 0.5-8 | coronary disease: 19 cases, hypertensive heart disease: 10 cases, rheumatic heart disease: 3 cases, dilated cardiomyopathy: 4 cases, pulmonary heart disease: 4 cases | / | 10/8 | 12/10 | SMI 100ml+WM | WM | 14 | 1,2 |
| Qu 2006 | 30/30 | 36/24 | T: 64.6±3.22  C: 63.8±3.18 | T: 5.26±1.56 C: 5.35±1.68 | coronary disease: 25 cases, hypertensive heart disease: 18 cases, rheumatic heart disease: 11 cases, pulmonary heart disease: 6 cases | / | 18/16 | 12/14 | SML 40m+WM | WM | 14 | 1,2,3,4,9 |
| Cui 2008 | 42/41 | 45/38 | 66.22 | 0.6-25 | coronary disease: 33 cases, dilated cardiomyopathy: 6 cases, chronic obstructive pulmonary disease: 12 cases,coronary disease with hypertension: 28 cases,others: 4 cases | 22 | 43 | 18 | SMI 8m/(kg· d), the maximum dose: 50ml/d | WM | 10 | 1,2,3,4 |
| Wang 2010 | 51/50 | 60/41 | T: 66.3±2.2 C: 65.7±2.3 | T: 4.8±2.3 C: 4.6±2.2 | coronary disease: 47 cases, hypertensive heart disease: 11 cases, dilated cardiomyopathy: 23 cases, valvular heart disease: 20 cases | 4/5 | 18/19 | 29/26 | SML 50ml+WM | WM | 15 | 1,2,3,4,6,9 |
| Tian 2010 | 31/28 | 36/23 | 66.32 | 0.6-20 | coronary disease: 30 cases, hypertensive heart disease: 13 cases, rheumatic heart disease: 6 cases, dilated cardiomyopathy: 10 cases | 12/10 | 14/15 | 5/3 | SML 40ml+WM | WM | 14 | 1,2 |
| Wu 2011 | 60/60 | 79/41 | 68±7.8 | … | coronary disease: 59 cases, hypertensive heart disease: 28 cases, dilated cardiomyopathy: 33 cases | / | 97 | 23 | SML 50ml+WM | WM | 14 | 1,2,9 |
| Liu 2011 | 40/40 | 55/25 | T: 55.2±6.8 C: 56.1±7.1 | T: 5-32 C: 5-30 | … | 6/7 | 16/16 | 18/17 | SML 40ml+WM | WM | 14 | 1,9 |
| Huang 2011 | 60/60 | 65/55 | T: 67.36±4.07 C: 69.27±3.96 | … | coronary disease: 54 cases, hypertensive heart disease: 49 cases, dilated cardiomyopathy: 17 cases | / | 47/48 | 12/13 | SML 50ml+WM | WM | 14 | 1,2,6 |
| Hu 2011 | 76/76 | 90/62 | T: 71.5±9.4 C: 72.3±8.6 | … | … | … | | | SML 50ml+WM | WM | 15 | 1,2,9 |
| Guo 2012 | 58/58 | 62/54 | T: 71.9 C: 72.7 | T: 3.6 C: 3.2 | … | 31/25 | 27/33 | / | SML 30ml+WM | WM | 15 | 1,2,5 |
| Pan 2014 | 63/63 | 71/55 | T: 67.3±11.5 C: 68.1±10.7 | T: 6.9±3.5 C: 6.5±4.1 | coronary disease: 52 cases, hypertensive heart disease: 27 cases, dilated cardiomyopathy: 47 cases | / | 24/27 | 39/36 | SMI 100ml+WM | WM | 7 | 1,6 |
| Hou 2014 | 40/40 | 47/33 | T: 67.1±4.3 C: 66.8±4.9 | … | coronary disease: 35 cases, hypertensive heart disease: 36 cases, dilated cardiomyopathy: 9 cases | / | 33/34 | 7/6 | SML 50ml+WM | WM | 14 | 1,2,6,9 |
| Ye 2015 | 30/30 | 26/34 | T: 64.4±8.8 C: 63.9±11.3 | T: 6.2 C: 6.6 | … | 11/13 | 11/14 | 6/5 | SML 60ml+WM | WM | 14 | 1,6,9 |
| He 2003 | 21/14 | 22/13 | T: 58.53 C: 60.23 | … | … | 4/3 | 12/9 | 5/2 | SQFZI 250ml+WM | WM | 14 | 1 |
| Yun 2006 | 55/51 | 54/52 | T: 65±7 C: 65±8 | … | coronary disease: 61 cases, hypertensive heart disease: 32 cases, dilated cardiomyopathy: 2 cases, pulmonary heart disease: 11 cases | … | | | SQFZI 250ml+WM | WM | 21 | 1,2 |
| Su 2009 | 40/40 | 47/33 | T: 67.7 C: 66.9 | T: 1.5-8 C: 2-7.5 | … | 13/14 | 27/26 | / | SQFZI 250ml+WM | WM | 14 | 1,9 |
| Liang 2009 | 43/38 | 57/24 | T: 67.38±3.54 C: 65.82±3.65 | … | coronary disease: 27 cases, hypertensive heart disease: 25 cases, rheumatic heart disease: 15 cases, ardiomyopathy: 14 cases | … | | | SQFZI 250ml+WM | WM | 14 | 1,3,4 |
| Wang 2011 | 32/31 | 38/25 | 62.0±10.4 | … | ischemic heart disease: 27 cases, hypertensive heart disease: 12 cases, dilated cardiomyopathy: 5 cases, pulmonary heart disease: 10 cases, valvular heart disease: 9 cases | … | | | SQFZI 250ml+WM | WM | 14 | 1,2 |
| Mao 2014 | 60/60 | 60/60 | 62.0±2.0 | … | coronary disease: 30 cases, rheumatic heart disease: 25 cases, hypertrophic cardiomyopathy: 35 cases, cardiopathy with hypertension: 30 cases | … | | | SQFZI 250ml+WM | WM | 28 | 1 |
| Wang 2014 | 120/120 | 167/73 | T: 65.32±8.36 C: 66.01±7.92 | … | coronary disease: 159 cases, hypertensive heart disease: 40 cases, dilated cardiomyopathy: 18 cases, pulmonary heart disease: 23 cases | 50/49 | 62/63 | 8/8 | SQFZI 250ml+WM | WM | 14 | 1,2,5,6,7,8 |
| Wu 2014 | 40/40 | 55/25 | T: 64.2±5.8 C: 65.3±5.0 | … | … | / | 24/26 | 16/14 | SQFZI 250ml+WM | WM | 21 | 1,6,7,8,9 |
| Liu 2015 | 26/26 | 30/22 | T: 61.4±7.2 C: 61.8±7.4 | T: 0.3-2 C: 0.3-2 | … | … | | | SQFZI 250ml+WM | WM | 21 | 1,2,3,4,7, |
| Wu 2015 | 40/39 | 39/40 | T: 58.31±12.30 C:57.42±11.68 | … | … | 23/20 | 14/15 | 3/4 | SQFZI 250ml+WM | WM | 15 | 1,2,3,4 |
| Yang 2015 | 31/32 | … | … | … | … | … | | | SQFZI 250ml+WM | WM | 14 | 1,2 |
| Chen 2017 | 29/29 | … | … | … | … | … | | | SQFZI 250ml+WM | WM | 14 | 1 |
| Wang 2014 | 35/29 | 25/39 | T: 54.9±7.4 C: 52.5±8.1 | T: 4.8±3.2 C: 5.4±3.8 | … | 9/7 | 20/18 | 6/4 | YQFMI 3.9g | WM | 14 | 1,2,3,7,8,9 |
| Yang 2014 | 60/60 | 24/96 | T: 72.2±4.3 C: 73.1±5.4 | … | … | 6/8 | 54/52 | / | YQFMI 5.9g | WM | 14 | 1,2 |
| Xue 2014 | 44/43 | 34/53 | 55.4±9.3 | … | … | 13 | 56 | 18 | YQFMI 2.6g | WM | 14 | 1,2,7 |
| Zhao 2015 | 76/72 | 91/57 | T: 65.88±11.23 C: 64.35±10.16 | … | … | … | | | YQFMI 3.9-5.2g | WM | 14 | 1,2,9 |
| Zhao 2015 | 30/30 | 35/25 | T: 70±4 C: 71±5 | … | … | 7/8 | 15/16 | 8/6 | YQFMI 2.6-3.9g | WM | 14 | 1 |
| Ren 2016 | 44/44 | … | 55±9.5 | … | coronary disease: 42 cases, hypertensive heart disease: 10 cases, rheumatic heart disease: 10 cases, dilated cardiomyopathy: 19 cases, pulmonary heart disease: 7 cases | 10 | 50 | 28 | YQFMI 5.2g | WM | 14 | 1,2,7 |
